# Risk factors for liver‐related mortality of patients with hepatitis C virus after sustained virologic response to direct‐acting antiviral agents

**DOI:** 10.1002/jgh3.12805

**Published:** 2022-08-26

**Authors:** Nobuhiro Hattori, Hiroki Ikeda, Tsunamasa Watanabe, Yosuke Satta, Takuya Ehira, Tatsuya Suzuki, Hirofumi Kiyokawa, Kazunari Nakahara, Hideaki Takahashi, Kotaro Matsunaga, Nobuyuki Matsumoto, Hiroshi Yasuda, Michihiro Suzuki, Fumio Itoh, Keisuke Tateishi

**Affiliations:** ^1^ Department of Internal Medicine, Division of Gastroenterology and Hepatology St. Marianna University School of Medicine Kawasaki Japan; ^2^ Division of Gastroenterology and Hepatology St. Marianna University Yokohama Seibu Hospital Yokohama Japan; ^3^ Division of Gastroenterology and Hepatology Kawasaki Municipal Tama Hospital Kawasaki Japan

**Keywords:** diabetes mellitus, direct‐acting antiviral agents, hepatitis C virus, survival, sustained virologic response

## Abstract

**Background and Aim:**

The aim of this study was to identify the factors associated with liver‐related and non‐liver‐related mortality of patients with hepatitis C virus (HCV) after sustained virologic response (SVR) to direct‐acting antiviral agents (DAAs).

**Methods:**

We conducted a retrospective, single‐center cohort study of HCV patients cured by DAAs.

**Results:**

A total of 330 patients with SVR to DAAs were eligible. The median follow‐up period was 3.38 years (inter‐quartile range: 2.03–4.58). The cumulative liver‐related or non‐liver‐related mortality rates at 1, 3, and 5 years were 0.00 or 1.29%, 2.87 or 3.60%, and 5.10 or 9.46, respectively. Among the liver‐related deaths, 9 of the 10 were from liver cancer. Among the non‐liver‐related deaths, the most common cause was malignancy. Through multivariate analysis using the Cox proportional hazard model, diabetes mellitus (DM, hazard ratio 13.1, 95% confidence interval 2.81–61.3) and a history of hepatocellular carcinoma (HCC, 12.8, 2.76–59.2), independently predicted liver‐related death. No variables were associated with non‐liver‐related death.

**Conclusion:**

Our findings suggest that DM and a history of HCC are risk factors for liver‐related mortality of HCV patients cured by DAAs. These results indicate that early management of HCV and HCC surveillance of diabetic patients after SVR are important to increase the chance of survival. Further studies are needed to confirm the association of DM and HCC history with survival.

## Graphical abstract

Very few studies have assessed the factors associated with liver‐related mortality in patients with sustained virologic response (SVR) following direct‐acting antiviral agents (DAA) treatment. The findings of our cohort study are that diabetes mellitus and a history of hepatocellular carcinoma (HCC) are risk factors for the liver‐related mortality of HCV patients cured by DAA. The results indicate that early management of HCV and HCC surveillance of diabetic patients after SVR are important to increase the chance of survival.

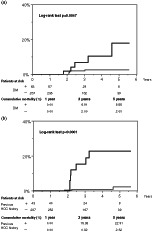

## Introduction

Hepatitis C virus (HCV) infection is a major cause of death worldwide, mostly through cirrhosis and hepatocellular carcinoma.^1^, ^2^ This serious global health problem can be improved by treating the infection with direct‐acting antiviral agents (DAA), which are highly effective and well tolerated. The risk of de novo hepatocellular carcinoma (HCC) in patients who achieve a sustained virologic response (SVR), through treatment with interferon or DAA, is known to be reduced.^3^, ^4^, ^5^, ^6^ Extrahepatic manifestations such as cryoglobulinemic vasculitis, lymphoma, cardiovascular diseases, and diabetes mellitus (DM) are recognized as a feature of HCV infection and can influence the morbidity, quality of life, and mortality of these patients. Several studies have shown a close link between viral eradication and a lower risk of extrahepatic manifestations, as well as an improvement of existing extrahepatic diseases.^7^ In addition, a recent assessment of the survival benefit of SVR by DAA confirmed decreases in all‐cause and liver‐related mortality.^8^, ^9^, ^10^, ^11^, ^12^ The French prospective cohort study showed that DAA treatment is associated with reduced risk for all‐cause mortality. Furthermore, that study reported a strong relation between all‐cause mortality and cirrhosis, markers of liver failure, and comorbidities (hypertension and anemia).^8^ Another single‐center study in Italy showed that liver‐ and non‐liver‐related death in HCV patients treated with DAA is strongly influenced by the patient's history before DAA treatment (HCC, variceal bleeding, and encephalopathy).^13^ However, very few studies have assessed the factors associated with liver‐related and non‐liver‐related mortality in patients with SVR following DAA treatment. The aim here was to identify those factors through a retrospective, single‐center cohort study.

## Methods

### Patients

We recruited patients with chronic hepatitis C who were administered IFN‐free DAA treatment between September 2014 and January 2021 at St. Marianna University Hospital. Among the patients with a history of HCC, those who had achieved a complete radiologic response to HCC treatment had received DAA treatment. Therefore, there was no patient who had active HCC at the time of initiation of the final DAA therapy. We excluded patients who were: (i) under the age of 18; (ii) co‐infected with human immunodeficiency virus or hepatitis B virus; (iii) had other chronic liver diseases, such as autoimmune hepatitis, primary biliary cirrhosis, and alcoholic liver disease, (iv) had no virological outcome (lost to follow‐up, withdrawal, death on treatment and so on); (v) failed their last DAA therapy; and (vi) had less the 90 days of follow‐up after initiation of DAA therapy. This study was conducted with the approval of the ethics committee of St. Marianna University School of Medicine, in accordance with the ethical standards laid down in the 1975 Declaration of Helsinki. The need for written informed consent was waived because of the retrospective nature of the study. The opt‐out method to obtain patient consent was used in our institution (Approval No. 4743).

### Patient characteristics at the time of initiation of the final DAA therapy

We collected data at the time of initiation of the final DAA therapy at St. Marianna University Hospital. Details of age, sex, HCV genotype, history of HCC, previous DAA treatment, DM, and hypertension were obtained from the medical records.

### Laboratory assessments

We collated the results of laboratory tests, such as aspartate aminotransferase (AST), alanine aminotransferase (ALT), albumin, total bilirubin, platelet, alpha‐fetoprotein (AFP), and mac‐2 binding protein glycan isomer (M2BPGi) before the final DAA treatment and at 12 weeks after the end of treatment.

The FIB‐4 index was calculated from the following equation: age (years) × AST (U/L)/{platelet counts (10^9^/L) × (ALT [U/L]) 1/2}.^14^

M2BPGi levels were measured as described previously.^15^ Briefly, quantification was via a lectin‐antibody sandwich immunoassay, conducted using an automated analyzer, HISCL‐5000 (Sysmex, Hyogo, Japan). M2BPGi levels were expressed as a cutoff index (COI), which was calculated according to the following formula: COI = ([M2BPGi]sample − [M2BPGi]negative control)/([M2BPGi]positive control − [M2BPGi]negative control).^16^

### Follow‐up after SVR

SVR was defined as undetectable serum HCV RNA, at week 12 after the end of treatment. All patients who achieved SVR were followed up every 3–6 months with measurements of blood cell counts, liver biochemistry, and tumor markers such as AFP. Ultrasonography or dynamic computed tomography (CT) with contrast media or gadolinium‐ethoxybenzyl‐diethylenetriamine pentaacetic acid‐enhanced dynamic magnetic resonance imaging (Gd‐EOB‐DTPA‐MRI) was also performed every 3–6 months. HCC was diagnosed using contrast media and imaging modalities, such as dynamic CT or Gd‐EOB‐DTPA‐MRI, or by histological evidence. Treatment of HCC was performed at St. Marianna University Hospital, based on Japanese guidelines.^17^ Patients who had been treated for HCC continued to be followed up every 3 months for the first 5 years after HCC treatment. According to the guideline, diabetic patients were managed by a diabetes specialist.^18^ Patients were followed up from the time of SVR to the last visit before the end of September 2021, and their outcomes were assessed using medical records.

### Statistical analysis

Data for continuous variables are expressed as the median (inter‐quartile range [IQR]). Data for categorical variables are expressed as frequency and percentage. The primary outcomes of this study were the incidence and predictors of liver‐related and non‐liver‐related death of HCV patients cured by IFN‐free DAA treatment. The start date of follow‐up is defined as the date of achieving SVR. We retrieved the cause of death from the medical records. Kaplan–Meier methods were used to identify the cumulative all‐cause, liver‐related, and non‐liver‐related mortality. Univariate and multivariate Cox proportional hazard models were used to identify the predictors of liver‐related and non‐liver‐related death. All *P* values for statistical tests were two‐tailed, and values of <0.05 were considered statistically significant. Statistical analyses were performed using JMP Pro 14.2.0 (SAS, Cary, NC, USA) and Prism 5 for Windows (GraphPad Software, La Jolla, CA, USA).

## Results

### Patients characteristics

**Figure 1:**
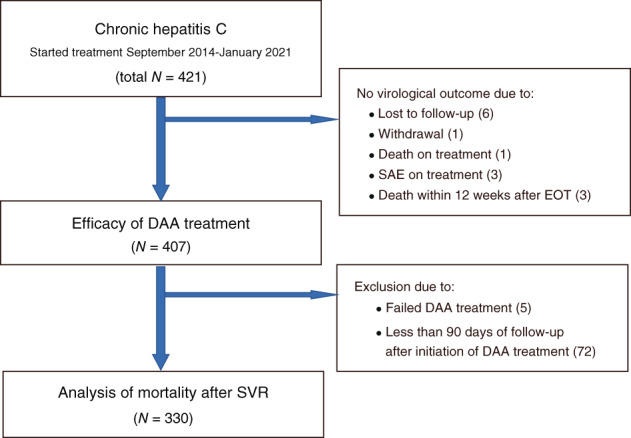
Study flowchart. DAA, direct‐acting antiviral agents; EOT, end of treatment; SAE, severe adverse event; SVR, sustained virologic response.

A total of 421 patients who started IFN‐free DAA therapy at St. Marianna University Hospital were identified. Fourteen of these patients were excluded because they lacked an SVR assessment. Of these, four patients died during the treatment period or within 12 weeks after the end of treatment. Death was attributable to liver failure in one patient, while the cause of death in the others was liver unrelated (Fig. 1). Of the 407 patients who had been evaluated for the efficacy of the last DAA treatment, 77 were excluded from analysis because they had failed DAA treatment (five patients) or had been observed for fewer than 90 days (72 patients).

Liver‐related and non‐liver‐related mortality was evaluated for 330 patients who achieved SVR and underwent follow‐up after treatment (Table 1). The median age at the time of final DAA initiation was 69 years and 128 (38.8%) were male. The majority (70.3%) were infected with HCV genotype 1. Sixteen patients (4.8%) had previous IFN‐free DAA therapy and 43 (13.0%) had a history of HCC treatment. Among 43 patients with a history of HCC treatment, HCC stages based on the Barcelona Clinic Liver Cancer staging system at the time of curative HCC treatment were 22 patients with stage 0, 19 patients with stage A, and one patient with stage B. In this cohort, 63 (19.1%) had diabetes and 131 (39.7%) had hypertension. The median FIB‐4 index was 2.80 at the time of initiation of the final DAA therapy.

**Table 1:** Demographic and clinical characteristics of the hepatitis C virus (HCV) patients treated with last direct‐acting antiviral agents (DAA)

Characteristics	*n* (%)
Age, years, median (IQR)	69 (61–75)
Gender: Male	128 (38.8)
HCV genotype	
1	234 (70.9)
2	94 (28.5)
3	2 (0.6)
Previous IFN‐free DAA treatment	16 (4.8)
Previous HCC treatment	43 (13.0)
DM	63 (19.1)
Hypertension	131 (39.7)
Platelet, ×10^4^/μL, median (IQR)	16.0 (11.8–19.8)
Serum albumin, g/dL, median (IQR)	4.1 (3.9–4.3)
Total bilirubin, mg/dL, median (IQR)	0.7 (0.6–0.9)
AST, U/L, median (IQR)	38 (27–55)
ALT, U/L, median (IQR)	36 (24–52)
FIB‐4 index, median (IQR)	2.80 (1.86–4.32)
AFP, ng/mL, median (IQR)	4.4 (3.0–7.8)
M2BPGi, COI, median (IQR)	1.77 (1.08–3.03)
Follow‐up period after SVR, years, median (IQR)	3.38 (2.03–4.58)

AFP, alpha‐fetoprotein; ALT, alanine aminotransferase; AST, aspartate aminotransferase; DAA, direct‐acting antiviral agents; DM, diabetes mellitus; HCC, hepatocellular carcinoma; HCV, hepatitis C virus; IFN, interferon; IQR, inter‐quartile range; M2BPGi, mac‐2 binding protein glycan isomer; SVR, sustained virologic response.

### Cumulative mortality after SVR

**Figure 2:**
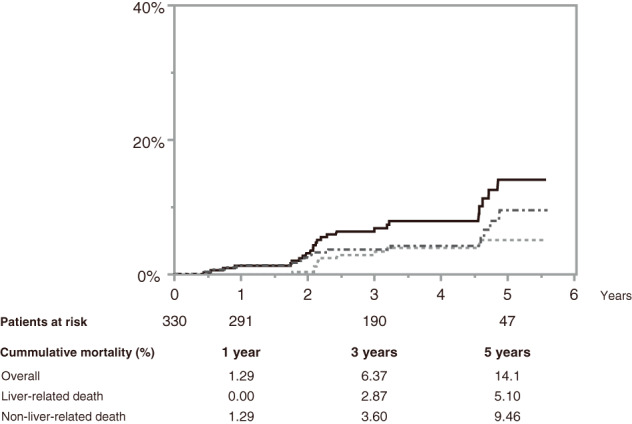
Cumulative incidence of overall liver‐related and non‐liver‐related mortality. The number of patients at risk is shown below at each time point: 1, 3, and 5 years. Cumulative mortality rates are also shown below at each time point. (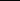), Overall; (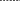), liver‐related death; (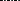), non‐liver‐related death.

For the SVR patients following DAA treatment, the median follow‐up period was 3.38 years (IQR 2.03–4.58). During 1481 person‐years of follow‐up, 25 patients died: 10 due to liver‐related death and 15 due to non‐liver‐related death. The cumulative all‐cause mortality was 1.29, 6.37, and 14.1% at 1, 3, and 5 years. The cumulative liver‐related and non‐liver‐related mortality rates were 0.00, 2.87, and 5.10% and 1.29, 3.60, and 9.46% at 1, 3, and 5 years, respectively (Fig. 2). Table 2 shows the causes of death. Of the liver‐related deaths, 9 of 10 cases were liver cancer. Among non‐liver‐related deaths, the most common cause of death was malignancy.

**Table 2:** Causes of death

	*n* (%)
All‐cause death	25 (100)
Liver‐related death	
Any	10 (40)
Liver cancer	9 (36)
Liver failure	1 (4)
Non‐liver‐related death	
Any	15 (60)
Malignancies	9 (36)
Cerebrovascular accident	3 (12)
Cardiovascular disease	2 (8)
Chronic pulmonary disease	1 (4)

### Variables associated with liver‐related and non‐liver‐related death

**Figure 3:**
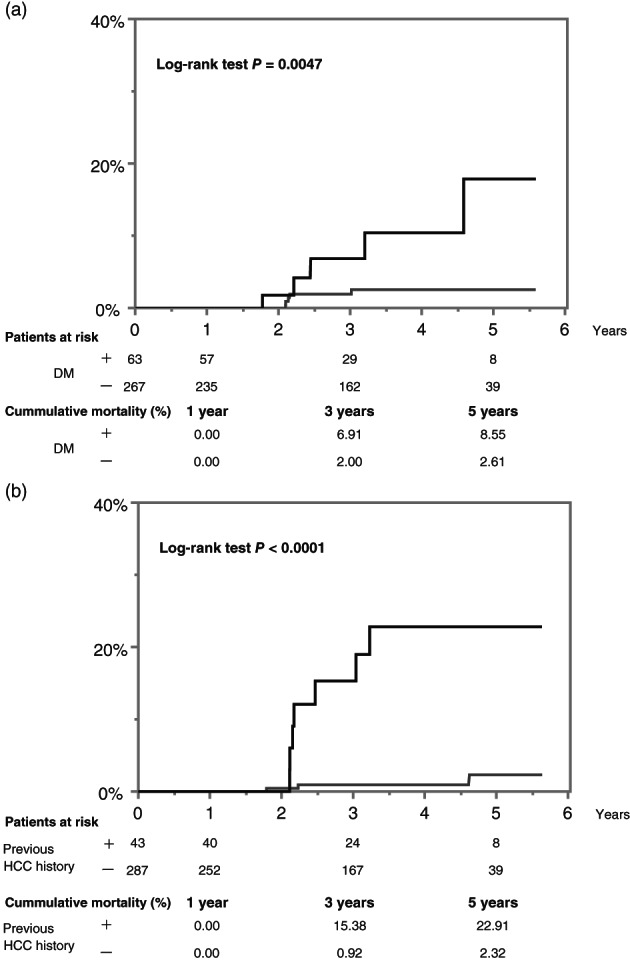
Cumulative incidence of liver‐related mortality for patients (a) with and without diabetes mellitus (DM) and (b) a history of hepatocellular carcinoma (HCC). The number of patients at risk is shown below at each time point: 1, 3, and 5 years. Cumulative mortality rates are also shown below at each time point. (a): (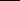), DM+; (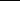), DM−. (b): (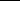), Previous HCC history+; (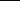), previous HCC history−.

In univariate analysis using the Cox proportional hazard model, DM, a history of HCC, pre‐FIB‐4 index, post‐FIB‐4 index, and post‐M2BPGi were associated with liver‐related death. In multivariate analysis, DM and a history of HCC independently predicted liver‐related death (Table 3). The 5‐year cumulative liver‐related mortality of SVR patients with and without DM was 8.55 and 2.61%, respectively (log‐rank test: *P* = 0.0047, Fig. 3a). The 5‐year cumulative liver‐related mortality of SVR patients with and without a history of HCC was 22.91 and 2.32%, respectively (log‐rank test: *P* < 0.0001, Fig. 3b). In addition, among the patients without a history of HCC, the 5‐year cumulative liver‐related mortality rates of SVR patients with and without DM were 13.7 and 0.00%, respectively (log‐rank test: *P* = 0.0001, Figure S1, Supporting information).

**Table 3:** Variables associated with liver‐related death in sustained virologic response patients following direct‐acting antiviral agents

		Univariate analysis		Multivariate analysis	
	Category	Hazard ratio (95% CI)	*P* value	Hazard ratio (95% CI)	*P* value
Age, years	Continuous	0.99 (0.95–1.06)	0.8527		
Gender	Male *versus* Female	2.69 (1.76–9.52)	0.1262		
Genotype	1 *versus* 2 or 3	1.48 (0.31–7.00)	0.6180		
DM	Yes *versus* no	5.01 (1.45–17.4)	0.0109	13.1 (2.81–61.3)	0.0010
Hypertension	No *versus* yes	1.67 (0.43–6.47)	0.4559		
History of HCC	Yes *versus* no	15.7 (4.06–60.8)	<0.0001	12.8 (2.76–59.2)	0.0011
Pre‐FIB‐4	Continuous	1.23 (1.03–1.43)	0.0022	0.86 (0.59–1.26)	0.4204
Pre‐AFP, ng/mL	Continuous	1.01 (0.97–1.03)	0.5208		
Pre‐M2BPGi, COI	Continuous	1.15 (0.96–1.34)	0.1199		
Post‐FIB‐4	Continuous	1.48 (1.17–1.81)	0.0022	1.38 (0.75–2.56)	0.3207
Post‐AFP, ng/mL	Continuous	1.02 (0.99–1.03)	0.1278		
Post‐M2BPGi, COI	Continuous	1.31 (1.13–1.47)	0.0017	1.17 (0.95–1.45)	0.1437

AFP, alpha‐fetoprotein; CI, confidence interval; DM, diabetes mellitus; HCC, hepatocellular carcinoma; M2BPGi, mac‐2 binding protein glycan isomer.

On the other hand, there was no variable associated with non‐liver‐related death (Table S1).

## Discussion

The findings of this single‐center Japanese cohort study are that DM and history of HCC are independently associated with an increased risk of liver‐related mortality in HCV patients with SVR following DAA treatment. These results indicate that early diagnosis and treatment of HCV are necessary to reduce liver‐related mortality. Currently, the American Association for the Study of Liver Diseases recommends HCC surveillance of patients with cirrhosis.^19^ Because the major cause of liver‐related death was HCC, adequate surveillance for HCC is needed in diabetic patients, regardless of the presence of cirrhosis.

A number of studies have analyzed the association between DM and HCC development in HCV patients and revealed that DM is an independent factor for an increased risk of HCC.^20^, ^21^, ^22^ According to the recent meta‐analysis conducted by Vancsa *et al*., DM increases the risk of HCC development after SVR following DAA treatment of HCV patients.^6^ In this manuscript, Vancsa *et al*. concluded that there is a two‐way association between HCV infection and DM. HCV patients had a high prevalence of DM. On the other hand, DM leads to the progression of inflammation, hepatic fibrosis, and oncogenesis in HCV patients.

The interrelationship between HCC and diabetes is incompletely understood. Nevertheless, hyperinsulinemia and oxidative stress are considered to be of prime significance in the progression of diabetes to cancer.^23^ Hyperinsulinemia has been suggested to be involved in carcinogenesis directly by promoting cancer initiation and progression, and indirectly through IGF‐1. Increased oxidative damage in diabetes is considered to be responsible for DNA damage, mutational changes in oncogenes, and eventually, cancer.^24^, ^25^

To our knowledge, three studies have established liver‐related death as a primary endpoint in HCV patients treated by DAA.^11^, ^12^, ^13^ Unlike this investigation, these studies failed to reveal a significant association between DM and liver‐related death. Of the three studies, two discussed the causes of death in detail and found that liver cancer caused 63.2% (12 of 19) and 51.9% (14 of 27) of liver‐related death. The remaining cause of death was liver failure.^11^, ^13^ In contrast, our results were that 9 of 10 patients with liver‐related death died of liver cancer and only one patient died of liver failure. Therefore, it is considered that the reason for the difference in the results of this and the previous studies is the difference in the cause of liver‐related death. Further studies are needed to confirm the association of DM with the reduced survival of patients with SVR after DAA treatment.

In this study, malignancy accounted for 60% of non‐liver‐related deaths. In contrast, according to 2019 demographic statistics published by the Ministry of Welfare, the death rate for malignancy was 27.3% in Japan. Our study included only 330 patients, and this small number may be the reason for bias in terms of the leading cause of death. In addition, the IQR of age of our study population was 61–75 years. In this age bracket, malignancy accounted for at least 40% of deaths, according to the vital statistics. This bias may have led to the discrepancy between our results and the vital statistics of the Ministry of Welfare.^26^

Most clinicians now consider DAA treatment to be the standard of care for patients with hepatitis C, even for those with a history of HCC.^19^ However, the association of a history of HCC and the probability of survival after SVR on DAA has not been discussed often. The Asian Pacific Association for the Study of the Liver, in its 2019 recommendations on follow‐up of DAA‐treated virus‐eradicated HCV‐infected patients, highlights a history of HCC and recommends a closer follow‐up of patients after treatment for HCV. In our study, we found that a history of HCC is associated with not only HCC recurrence but also liver‐related mortality.^27^ This result confirms the correctness of a closer follow‐up of patients with a history of HCC. In addition, patients with a history of HCC often have been excluded from such studies.^8^, ^9^, ^11^ An Italian study included patients with a history of HCC and evaluated the association between the variables at the time of initiation of the final DAA therapy and liver‐related death. In that study, a significant association of liver‐related mortality with a history of HCC was observed by univariate analysis, but was not detected by multivariable analysis.^13^ Further studies are also needed to reveal the impact of a history of HCC on survival.

Nakagawa *et al*. evaluated the relationship between all‐cause mortality and M2BPGi levels at the time of SVR and reported that a high M2BPGi level at the time of SVR was associated with an increase in all‐cause mortality.^28^ In our study, in univariate analysis, pre‐FIB‐4 index, post‐FIB‐4 index, and post‐M2BPGi were associated with liver‐related death. However, pre‐M2BPGi was not associated with liver‐related death. These results suggested that not pre‐M2BPGi but post‐M2BPGi affect survival of patients with SVR after DAA treatment. In multivariate analysis, there was not a statistically significant association between these variables and survival of patients with SVR after DAA treatment. Nakagawa *et al*. analyzed predictors associated with all‐cause mortality in patients without a history of HCC. Meanwhile, in our study, the predictors associated with death were analyzed separately and divided into liver‐related death and non‐liver‐related death, regardless of a history of HCC. Thus, it is suggested that the different methods of analysis had led to different outcomes.

In the Italian cohort, Calvaruso *et al*. investigated the predictors of liver‐related and cardiovascular mortality after DAA treatment using Cox proportional hazards regression analysis and found that SVR had the most impact on cardiovascular mortality after DAA treatment (hazard ratio, 0.07; *P* < 0.01). This study also showed that diabetic patients had a 3.45‐fold increased risk of cardiovascular death (*P* = 0.014).^11^ In our cohort, we analyzed the factors associated with non‐liver‐related death. However, no variable was significantly associated with non‐liver‐related death. The fact that only 3 of the 15 patients with non‐liver‐related death had cardiovascular disease as the cause may have affected this result.

Our study has several limitations. First, the sample size is small and we might not have been able to demonstrate a statistically significant difference in some variables using multivariable analyses. Second, other variables linked to HCC development, including history of obesity and alcohol intake, should have been collected as comprehensively as possible from our patients.^29^ Third, the follow‐up period might be considered short for assessing patient mortality.

In conclusion, the findings of our cohort study are that DM and history of HCC are risk factors for the liver‐related mortality of HCV patients cured by DAA. The results indicate that early management of HCV and HCC surveillance of diabetic patients after SVR are important to increase the chance of survival. Further studies are needed to confirm the association of DM and history of HCC with survival.

## Supporting information


**Figure S1.** Cumulative incidence of liver‐related mortality in 284 patients without a history of HCC according to presence or absence of DM.
**Table S1.** Variables associated with non‐liver‐related death of patients with SVR following treatment with DAA.
