# Supplementary material for: Risk factors for liver‐related mortality of patients with hepatitis C virus after sustained virologic response to direct‐acting antiviral agents
Source: JGH Open. 2022 Aug 26;6(10):685–91. doi: 10.1002/jgh3.12805 (PMC9575322; doi:10.1002/jgh3.12805)
Supplement: Supplementary file 1 — Figure S1. Cumulative incidence of liver‐related mortality in 284 patients without a history of HCC according to presence or absence of DM. Table S1. Variables associated with non‐liver‐related death of patients with SVR following treatment with DAA. [file JGH3-6-685-s001.docx]

**Risk factors for liver-related mortality of patients with hepatitis C virus after sustained virologic response to direct-acting antiviral agents**

Nobuhiro Hattori, Hiroki Ikeda, Tsunamasa Watanabe, Yosuke Satta, Takuya Ehira, Tatsuya Suzuki, Hirofumi Kiyokawa, Kazunari Nakahara, Hideaki Takahashi, Kotaro Matsunaga, Nobuyuki Matsumoto, Hiroshi Yasuda, Michihiro Suzuki, Fumio Itoh and Keisuke Tateishi

Table of contents

Supplementary Figure 1 ・・・・・・・・・・・・・・・・・・・・・・・・・・・・・・・・・・・・・・・・・・・・・・・・・・・・・・・・　2

Supplementary Table 1 ・・・・・・・・・・・・・・・・・・・・・・・・・・・・・・・・・・・・・・・・・・・・・・・・・・・・・・・・・　3

Supplementary Figure 1. Cumulative incidence of liver-related mortality in 284 patients without a history of HCC according to presence or absence of DM.


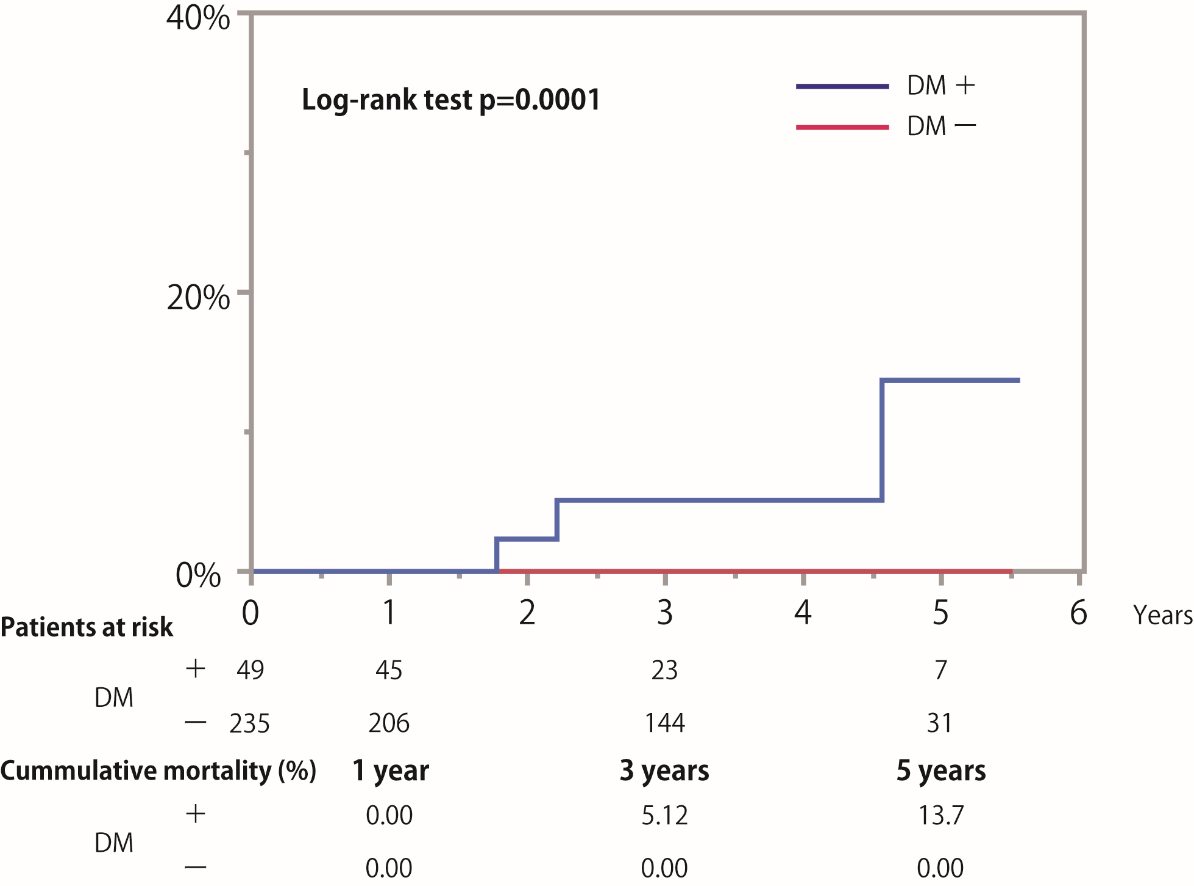


The number of patients at risk are shown below at each time point, 1 year, 3 years and 5 years. Cumulative mortality rates are also shown below at each time point.

Supplementary Table 1. Variables associated with non-liver-related death of patients with SVR following treatment with DAA.

|  | | Univariate analysis | | Multivariate analysis | |
| --- | --- | --- | --- | --- | --- |
|  | Category | Hazard  Ratio (95% CI) | P value | Hazard Ratio (95% CI) | P value |
| Age, y | Continuous | 1.04 (0.99-1.12) | 0.0806 |  |  |
| Gender | Male vs. Female | 2.07 (0.75-5.73) | 0.1593 |  |  |
| Genotype | 1 vs. 2 or 3 | 2.01 (0.71-5.71) | 0.1904 |  |  |
| DM | Yes vs. No | 1.73 (0.55-5.44) | 0.3514 |  |  |
| Hypertension | No vs. Yes | 1.60 (0.58-4.41) | 0.3650 |  |  |
| History of HCC | Yes vs. No | 1.03 (0.23-4.58) | 0.9669 |  |  |
| Pre-FIB-4 | Continuous | 1.08 (0.89-1.25) | 0.3931 |  |  |
| Pre-AFP, ng/ml | Continuous | 1.00 (0.97-1.02) | 0.7296 |  |  |
| Pre-M2BPGi, COI | Continuous | 1.13 (0.95-1.29) | 0.1491 |  |  |
| Post-FIB-4 | Continuous | 1.18 (0.92-1.42) | 0.1729 |  |  |
| Post-AFP, ng/ml | Continuous | 1.01 (0.96-1.03) | 0.4998 |  |  |
| Pre-M2BPGi, COI | Continuous | 1.02 (0.61-1.28) | 0.9167 |  |  |

Abbreviations: AFP, alpha-fetoprotein; DAA, direct-acting antiviral agents; DM, diabetes mellitus; HCC, hepatocellular carcinoma; M2BPGi, mac-2 binding protein glycan isomer; SVR, sustained virological response.
